# Ingenious Quackery

**Published:** 1888-10

**Authors:** 


### INGENIOUS QUACKERY.

The modern quack is plausible and ingenious. A short time ago one of the fraternity experimented in Lambeth, England, with considerable success upon the pockets of an awe-stricken crowd. After a preliminay harangue and a terse little lecture on the viscera, which the charlatan sketched in with colored crayons upon a blackboard on which the human skeleton was outlined in white paint, the fellow came to business. "I am going to demonstrate to you," said he, "by a startling experiment upon one of you bystanders that my miraculous remedy can cure all diseases of the lungs and chest. Now, whoever's got a bad cough or cold on the chest, let him stand forward." There was some little hesitation and a good deal of giggling. "Don't be afraid, my friends," said the quack; "its all free, gratis for nothing. Let any afflicted person come forward and I'll show him the nature of his disorder and give him a packet of my lung healers for nothing." At last a man with a violent cold and cough came forward. The quack doctor pretended to sound his chest with a stethoscope of almost pantominic proportions, and then informed the staring crowd that the patient was in a galloping consumption. "My friend," said the quack to the unfortunate victim, "so terrible is this disease that you can actually see it." He handed a glass tube to the patient and then poured a pint of clear water into a large tumbler, "Just you blow into that water, my friend," he cried. The man obeyed, and the water grew discolored, turbid, and at last as white as if it had been mixed with milk. The patient himself became as pale as ashcs. "This unhappy man, my friends," said the quack as he held the glass on high, "if he hadn't had the good fortune to come across me to-night wouldn't have been long for this world. I should have given him about a fortnight. That's all. Now, a packet of my lung healers will cure him. What you see in the glass of water are his vitiated humors, the product of corruption. My magic lung healers destroy these humors in the body or out of the body. Observe, my friends, watch me carefully; there is no deception here." The quack dropped a pinch from one of a packet of powders into a glass, and directed the patient to stir it with the tube. The water became immediately clear. Then he reaped his harvest. The explanation is, of course, very simple. The water was lime water, and the carbonic acid in the man's breath naturally threw down the carbonate of lime at once, and rendered the water turbid. And the miraculous lung healer was simply a little citric acid and sugar, which instantly redissolved it.—*Pacific Record.*

